# Understanding the factors influencing healthcare providers’ burnout during the outbreak of COVID-19 in Jordanian hospitals

**DOI:** 10.1186/s40545-020-00262-y

**Published:** 2020-09-22

**Authors:** Abdullah Algunmeeyn, Faris El-Dahiyat, Mohammad Mitib Altakhineh, Mohammad Azab, Zaheer-Ud-Din Babar

**Affiliations:** 1 School of Nursing, University of Isra, Amman, Jordan; 2 College of Pharmacy, Al Ain University, Al Ain, UAE; 3 Karak Comprehensive Medical Center, Jordanian Ministry of Health, Amman, Jordan; 4 Faculty of Medicine, The Hashemite University, Zarqa, Jordan; 5 Department of Pharmacy, University of Huddersfield, Huddersfield, UK

**Keywords:** Pharmacists, Physicians, Nurses, Healthcare providers, Burnout, COVID-19, Factors, Jordan, Hospital, Qualitative

## Abstract

**Abstract:**

**Background:**

Coronavirus disease (COVID-19) is an infectious disease caused by a newly discovered coronavirus. The nature of healthcare providers’ occupation puts them at an increased risk of getting any contagious disease, including COVID-19. They are on the front line of the COVID-19 outbreak response and as such are at risk of contracting this virus. The infectious disease started from China in December 2019 and spread rapidly throughout countries, including Jordan. Especially, recent studies indicated that Jordanian healthcare providers’ work conditions and demographic are significant factors for healthcare providers’ burnout. Additionally, burnout has been increased among healthcare providers in Jordanian hospital.

**Aim:**

The present investigation aims to better understand the factors affecting pharmacists’, physicians’, and nurses’ burnout during the outbreak of COVID-19 to provide basic information for lowering and preventing the level of burnout in Jordanian hospitals.

**Method:**

This study is qualitative in nature, adopting face-to-face interviews as the key instrument of data collection in one hospital in Jordan. The sample interviewed consisted of 30 healthcare providers in total (10 nurses, 10 physicians. and 10 pharmacists).

**Result:**

Three key factors to healthcare providers’ burnout were identified in the sampled hospitals: job stress, staff and resource adequacy, fear of COVID-19 infection, and interprofessional relationships in healthcare practice. The examination also offers recommendations for lowering and preventing healthcare providers' burnout in Jordanian hospitals.

**Conclusion:**

This study explored the main factors of healthcare providers’ burnout during the outbreak of COVID-19 in Jordanian hospitals, thereby making an original contribution to existing knowledge, as it is the first empirical exploration of healthcare providers' burnout during the outbreak of COVID-19. As such, it has attempted to offer an in-depth understanding of the factors impacting this issue.

## Background

The novel virus was first identified by Chinese authorities in Wuhan City at the end of December 2019. The evidence presented shows that the virus is spread through birds and mammals, with people particularly at risk of infection and spread of the virus [1]. COVID-19 is similar to the previous outbreaks of corona viruses such as SARS-CoV (severe acute respiratory syndrome-coronavirus) and MERS-CoV (Middle East respiratory syndrome-coronavirus) in 2003 and 2015 [2]. The infection started to spread rapidly throughout countries, including Jordan. In December of 2019, the China Health Authority alerted the World Health Organization (WHO) to a serious case of pneumonia that was unknown to doctors and that was occurring in Wuhan, China [3, 4]. The infection started to spread rapidly to other countries including Jordan. It seems that the rapidly spreading virus is more contagious than SARS-CoV and MERS-CoV9 [4].

A suggested route of human-to-human transmission is through airborne droplets, contact or touching an infected person, or a contaminated surface [5]. The nature of healthcare providers’ occupation puts them at an increased risk of getting any contagious disease, including COVID-19. They are on the front line of the COVID-19 outbreak response and as such are at risk of contracting this virus. Hand washing with soap and water, wearing face masks, and isolating confirmed and suspected cases are considered the best ways to avoid being exposed to COVID-19 [6, 7]. However, a nationwide outbreak of COVID-19 is an unexperienced virus for healthcare providers (nurses, doctors, and pharmacists) in the world. Similarly, when severe acute respiratory syndrome such as SARS spread rapidly, most healthcare providers experienced severe stress, and some nurses refused to care for patients [8, 9]. For example, the factors influencing nurses’ desire to leave their jobs during an outbreak of SARS were identified as the perceived risk of fatality from SARS, tenure, work stress, and social relationships.

Burnout is a long-term consequence of prolonged exposure to certain job demands and a reaction that appears when a person can no longer endure the stress they have been undergoing [10]. Additionally, burnout is a syndrome characterized by emotional exhaustion, depersonalization, and a diminished sense of personal achievement. Both quantity and quality of healthcare providers’ care could be negatively affected by stress and burnout. Evidence found that stressors in the workplace may result in a burnout syndrome and burnout results in low productivity [11]. Studies reported that healthcare providers are faced with hectic, unpredictable, and ever-changing situations [11, 12]. For the reason that they deal with various diseases, traumatic events, and urgent situations, they do not have enough time for recovery, putting them under persistent stress. The COVID-19 pandemic is causing global panic [13]. As a result, healthcare providers who were caring for COVID-19 patients are reported to experience much higher stress, anxiety, and burnout than healthcare providers who do not in other hospital departments [11]. A nationwide outbreak of COVID-19 is an unfamiliar traumatic event for healthcare providers; therefore, there has been limited studies into COVID-19 related burnout. Likewise, when severe acute respiratory syndrome such as SARS and MERS-CoV spread rapidly, most physicians, nurses, and pharmacists experienced severe anxiety, stress, and some physicians, and nurse declined to provide care for patients [8, 9]. Moreover, a study conducted by Tzeng and Yin [14] reported that when H5N1 avian flu spread rapidly, the healthcare providers' fear of infection from the disease was a significant factor influencing their preparedness to offer care for patients infected with the avian flu. Since an infectious disease such as COVID-19 can occur anywhere in the world, policy-makers and managers need to pay attention to healthcare providers’ burnout in association with their experiences of a nationwide COVID-19 outbreak. Especially, recent studies indicated that Jordanian healthcare providers’ work conditions and demographic are significant factors for healthcare providers’ burnout [15, 16]. Additionally, a study conducted by Alfuqaha and Alsharah [16] found that burnout has been increased among healthcare providers in Jordanian hospital. However, as yet, there is no research regarding these matters in relation to COVID-19-related burnout. Thus, this study attempted to recognize the influencing factors on healthcare providers’ burnout during an outbreak of COVID-19 in Jordanian hospitals to provide basic information for lowering and preventing the level of burnout.

## Methods

### Research design

The current project was qualitative in nature. It employed face-to-face interview (semi-structured interviews) as a method to gather data within one governmental hospital in Jordan.

### The study sample

The population for this qualitative study consisted of nurses, physicians, and pharmacists who were caring for patients’ suffering from COVID-19 admitted in one hospital located in a region Jordanian’s capital, Amman. The study participants were 10 nurses, 10 physicians, and 10 pharmacists who worked in the governmental hospital with the following job titles: pharmacists, registered nurse, and physicians who worked in the emergency department and intensive care unit. The selection of participants for the interviews was based on convenience and purposive sampling technique, resulting in a total of 30 participants (see Table 1). The prime consideration with the sampling process in this case was to recruit and select informants who could provide a deep understanding of the issues under investigation and to recruit and select them in such a way as to give credibility to the findings so that they can be potentially generalized to another context or a larger population.

**Table 1:** Numbers of interviewees in the case study

Staff category	Number of interviewees in case study
Pharmacists	**10**
Nurses	**10**
Physicians	**10**

Seven of the participants were females, and all the other participants were males, also working in different positions. Out of all those approached to be interviewed, no one declined to take part. Privacy and confidentiality were assured by the researcher to all the participants. For privacy and confidentiality reasons, the names of the interviewees cannot be mentioned. Participants were referred to using a code, consisting of H and numbered 1–30 in abbreviated format; for example, “Interviewee H1” or “Interviewee H10.” The selection of hospital healthcare providers for the interviews was based on convenience and purposive sampling technique as mentioned previously, resulting in a total of 30 respondents. The prime consideration with the sampling process was to recruit and select informants who could offer a deep understanding of the matters under examination and to recruit and select them in such a way as to give credibility to the results so that they can be potentially generalized to another context or a larger population.

### Data gathering

Face-to-face interviews were conducted to gather data from the two hospitals in this investigation, which started after gaining the essential ethical approval. Any participants who were willing to be interviewed were then sent an email form, indicating that they had agreed to take part and giving an opportunity for the investigator to arrange an appointment for the interview. Consent forms and information sheets were then distributed to the prospective participants based on their interest in participating in this study. The participant information sheet contained details of the investigation study, and the approaches that would be utilized to gather and analyze the relevant data, as well as the purposes for which these data would be used. Likewise, the participants were informed that they had the right to withdraw from the study at any time. In addition, the researcher asked the participants to sign a consent form on the day of the interview, emphasizing that their needs took priority over the study. Their permission was likewise obtained for the interview to be audio-recorded and for future use of the transcripts. Each interview was then digitally recorded, while notes were also taken during the interviews. The face-to-face interview process began with greetings and an introduction, after which the participant read through the information sheet and signed the informed consent form. The interviewee’s background data and demographic information were subsequently collected, followed by the scheduled interview questions and prompts.

### Data analysis

The data from the 30 interviews were analyzed with the aid of the NVivo qualitative data analysis software package. The audio-recorded interviews were transcribed verbatim, and these transcripts were reviewed by the researchers before the data within them was analyzed. Later, the data were imported into the NVivo qualitative data analysis software package to assist with linking concepts contained in the data. In this study, thematic analysis was selected as the data analysis method, since it was considered as the most appropriate method for the current study. This technique adopted in the present study was to place the mass of qualitative data collected into meaningful and related categories. This allowed the investigators to rearrange and analyze the data systematically and rigorously. In addition, thematic analysis enables rich and deep insights to be gained of intricate phenomena. The data analysis procedures for this study were as follows: (1) the researcher familiarized himself with the content and information in the transcriptions; (2) the researcher continued to read each transcription line by line to find keywords, meanings, or concepts relating to the aims of the study; (3) the coding was refined by assembling similar codes and attempting to group codes together; (4) in the practical retrieval process, the researcher collected in one place all the text that was coded with the same code, copying and pasting the text into separate labeled files for each code, and using the Word program; (5) the researcher organized and gathered all coded data into themes; and (6) common themes were identified and inferences were made from them, with sample quotes being selected to illustrate the themes that emerged.

## Results

### Characteristics of the interviewees

Only 7 of the participants were female. The majority of the interviewees were over 27 years of age, and most were graduates. They worked in different roles, but all had over 4-year work experience, while half had 12–18-year experience. A brief profile of each interviewee is presented in Table 1, 2, 3 and 4. Only 7 of the participants were females, and these consisted of 10 nurses, 10 doctors, and 10 pharmacists. All the other interviewees were males also working in different positions. The age of the participants ranged from 27 to 56, with the majority falling into the 33–42 age group (see Table 1, 2, 3 and 4). Their qualifications differ: 3 with diploma degrees, 22 with bachelor’s degrees, 4 with Pharm D degree, and 1 with Master degree.

**Table 2:** Participant profiles: nurses

No.	Designation	Gender	Age	Qualifications	Years of experience
1	ICU nurse	Male	30	Bachelor	8
2	ICU nurse	Male	33	Bachelor	11
3	ICU nurse	Male	29	Bachelor	8
4	ICU nurse	Female	29	Diploma	8
5	Emergency department nurse	Male	40	Bachelor	18
6	Emergency department nurse	Female	38	Bachelor	16
7	Emergency department nurse	Female	28	Bachelor	6
8	Emergency department nurse	Female	42	Bachelor	20
9	Emergency department nurse	Male	38	Diploma	18
10	Emergency department nurse	Male	40	Diploma	18

**Table 3:** Participant profiles: physicians

No.	Designation	Gender	Age	Qualifications	Years of experience
1	Ear, nose, and throat physician	Male	40	Bachelor	16
2	Pathologist and laboratory physician	Female	58	Bachelor	34
3	Radiologist	Male	56	Bachelor	32
4	Critical care practitioner physician	Male	44	Bachelor	20
5	Critical care practitioner physician	Male	41	Bachelor	21
6	Radiologist	Male	35	Bachelor	11
7	Surgeon	Male	39	Bachelor	15
8	Pathologist and laboratory physician	Male	42	Bachelor	18
9	Critical care practitioner physician	Male	33	Master	9
10	Surgeon	Male	55	Bachelor	31

**Table 4:** Participant profiles: pharmacist

No.	Designation	Gender	Age	Qualifications	Years of experience
1	Clinical pharmacist	Male	40	Bachelor	17
2	Clinical pharmacist	Female	45	Bachelor	22
3	Clinical pharmacist	Male	33	Bachelor	10
4	Clinical pharmacist	Male	29	Pharm D	6
5	Clinical pharmacist	Male	27	Pharm D	4
6	Clinical pharmacist	Male	31	Bachelor	8
7	Clinical pharmacist	Female	39	Bachelor	16
8	Clinical pharmacist	Male	35	Bachelor	12
9	Clinical pharmacist	Male	27	Pharm D	4
10	Clinical pharmacist	Male	30	Pharm D	7

Out of all those approached to be interviewed, no one declined, citing a lack of time or other commitments as a reason. The examiner decided to interview the staff who provided care for patients suffering from COVID-19 in the respective categories by concentrating on the nurses, doctors, and pharmacists in each category, as they would have had knowledge of the factors influencing healthcare providers’ burnout during the outbreak of COVID-19. The interview questions were designed to collect in-depth information on the following key areas that are factors influencing healthcare providers’ burnout during the outbreak COVID-19.

### Themes related to the factors influencing healthcare providers’ burnout during an outbreak of coronavirus in Jordanian hospitals

The data analysis revealed the following key themes relating to the factors influencing healthcare providers’ burnout during an outbreak of COVID-19 in hospitals in Jordan, as described by the participants during the interviews.

#### Job stress among healthcare providers

The data analysis revealed that most of the interviewees cited job stress as the key influencing factor of COVID-19. All of the participants (nursing, physicians, and pharmacists) mentioned fear and anxiety. Interviewees felt they did not have enough information when they were caring for patients, and they were afraid of infection spreading to their families. Thus, they became stressed and afraid. The following statements reflect the interviewees’ views in relation to this finding (theme):

... I am expected to be there and stay there and provide care for those patients suffering from COVID-19, but know that I am at risk and know that I have a family to go home to,. (Interviewee H15)

but . I have a husband and two children. The younger one is four years old and the little one is two years old. It is the time for them immunity wasn’t too strong, so I have to consider it very seriously. But what do you think I could I do? I have to be involved as a nurse unless I loss my job. (Interviewee H1)

Some COVID -19 patients are in this hospital. Since of the responsibility of my job, the pressure is inescapable for me (Interviewee H2)

As a nurse, I have a task to care for patients but at the same time I also have a responsibility to myself, my kids', wife and my parents, everybody who has a stake in my wellbeing. (Interviewee H3)

as you know we heard that the deaths of several pharmacists, nurses and doctors in around the world. Because we are work at the frontline to fight this virus so this cause fear and anxiety to get this virus or transit it to our families. (Interviewee H27)

#### Staff and resource adequacy

The data analysis found that most of the respondents emphasized staff and resource adequacy and turnover as a factor impacting nursing, physicians’, and pharmacists’ practice during an outbreak of COVID-19 at Jordanian hospitals. These opinions were derived from the following quotes extracted from the interviews with staff:

... I our hospital provide the all staff work with patient have COVID-19 adequate PPE such N95s and full Hazmat. (Interviewee H18)

... Our hospital employs the best infection control guideline for preventing the spread of COVID-19… (Interviewee H26)

...and I also see the shortage of nurses as a major barrier to Accreditations implementation in this hospital, because nursing is the area of healthcare provision where there is highest engagement and commitment to Accreditations implementation in this hospital. (Interviewee H1)

The staff sufficiency in this hospital leads to decrease workload for current staff and surely affects our performance and the quality of care provided to patients with COVID-19...... Management of our hospital is equipped with facilities sufficient for decrease or preventing the staff from infected from COVID-19the spread of COVID-19, (Interviewee H16,H22)

Our hospital employs the best infection control guideline for preventing the spread of COVID-19 between the staff , (Interviewee 14)

#### Fear of COVID-19 infection

The study found that the respondents recognized fear of COVID-19 infection as a factor influencing nursing, physicians, and pharmacists’ practice during an outbreak of COVID-19 in Jordanian hospitals. For example, the interviewees stated

In spite of we used good equipment to prevent our self during working with patient COVID-19 but honestly I am afraid of being infected with COVID-19. (Interviewee H3)

……….always pharmacists and doctors are bringing home bacteria and viruses. (Interviewee H5)

….I feel that my job puts me, wife and my son at great risk get the infection (interviewee H8)

I tried to protect myself as much as I can by the wearing Hazmat suit and follow the best infection control guideline for preventing myself to infected by COVID-19. I have a husband and two children. The younger one is four years old and the little one is two years old. It is the time for them immunity wasn’t too strong, so I have to consider it very seriously. But what do you think I could I do? I have to be involved as a physician unless I lose my job. (Interviewee H30)

## Discussion

This study aimed to explore the factors impacting healthcare providers’ burnout during an outbreak of COVID-19 in Jordanian hospitals in order to gain a better understanding of such influencing factors in hospitals in Jordan, specifically from the perspective and experience of nursing, physicians, and pharmacist staff. The respondents, including nurses, cited job stress, staff and resource adequacy and fear of COVID-19 infection as factors impacting the burnout in healthcare providers who experienced COVID-19 in Jordanian hospitals.

The findings show that job stress among healthcare providers was the main factor for burnout during the outbreak of COVID-19 in Jordanian hospitals. Most of the participants believe that during the outbreak of a new emerging contagious disease COVID-19 led to an increase of the number of affected patients with COVID-19 in their hospitals. This could increase the workload and expose them at work to COVID-19, the highly contagious nature of which could mean taking the infection home to their relatives. This result is similar to that of other studies, conducted by Shanafelt et al. and Potter [5, 17], where it was confirmed that the highly infectious nature of the disease increased healthcare providers’ stress and anxiety, which in turn, increased their burnout.

Additionally, according to the participants’ responses, the staff and resource adequacy was recognized as a factor in the current context. Most of the participants made the point that staff and resource adequacy in the hospitals was largely connected to the healthcare providers’ burnout, including reference to available and sufficient personal protective equipment, quick access to occupational health with efficient assessment and testing if symptoms warranted it, and information and resources to prevent carrying the infection home to family members. A study mentioned that hospitals usually provide personal protection equipment for doctors, nurses, little attention is being paid towards the protection for pharmacists even though they are the front line of fighting COVID-19 crisis [18, 19]. The current study also found that where there was an adequate increase in pharmacist, nurse, and physician staff in hospitals, this was associated with a decrease in healthcare providers’ burnout. Hospitals that invest in more staff may also invest in other activities that improve quality and avoid workload. These findings are consistent with those of other studies by Kim et al. and Kane et al. [20, 21], who highlighted such factors as being associated with the healthcare providers’ burnout during the outbreak of COVID-19. For example, they pointed out that MERS-CoV-related healthcare providers’ burnout are associated with poor hospital resources for the prevention of their staff contracting the virus and treatment of MERS-CoV. Additionally, Kane et al. [22] and Klopper et al. [23] found that nurse-to-patient ratio is an effective nurse retention strategy leading to nurses’ satisfaction with the job and providing care and decreasing nurses’ burnout. Furthermore, a study indicated that management should ensure presence of adequate human resource particularly pharmacists to decrease the workload. This can be attained through recruiting new staff and increasing efficiency [24].

Finally, the findings of this project revealed that fear of COVID-19 infection is a factor related to healthcare providers’ burnout in Jordanian hospitals. The interviewees believe that nursing, physician, and pharmacist jobs put the healthcare providers and their families at great risk of exposure to COVID-19 due to acquiring the infection at work and taking the infection home to their family. This finding is supported by those of other studies conducted worldwide [5, 9, 14, 25–27].

The principles of COVID-19 infection prevention and control strategies associated with healthcare suggest the need for administrative controls and hospital resources [27]. In addition, if the government, with strong leadership, develops stronger and more resilient health systems in preparation against emerging contagious diseases such as COVID-19, healthcare provides’ burnout will decrease.

## Conclusions and recommendations

The present study has explored the main factors affecting healthcare providers’ burnout, as faced by hospitals in Jordan during the outbreak of COVID-19, based on responses from a sample of hospital pharmacists, physicians and nurses’ staff members. In Jordanian hospitals, three main factors were identified from the reported experiences of these nurses: job stress among healthcare providers, staff and resource adequacy, and fear of COVID-19 infection. These factors should be addressed, because they could impact on healthcare providers’ burnout in hospitals, negatively influencing the quality of healthcare services and thereby incurring serious problems.

Nevertheless, a limitation of the current project is that it examined just one hospital. Moreover, the sample only included pharmacists, physicians, and nurses caring for patients of coronavirus in the emergency department and intensive care unit, which is likely to affect the generalizability of the findings. Further enquiry is therefore necessary to explore the ways in which the factors affect the healthcare providers’ burnout during an outbreak of COVID-19 in Jordan’s and government hospitals, thereby looking at how to decrease and prevent staff burnout to identify and overcome these factors, with the inclusion of views from managers and policy-makers.

In order to lower the level of burnout, managers need to make efforts to reduce job stress, to reinforce hospital resources for the treatment of COVID-19, and to promote support from top management. And also, sufficient funds are available to meet all of quality of care needs of the patient in the hospital. And being able to meet requirement or at least minimum requirement (adequate physicians, pharmacists, and nurse-patient ratio, enough equipment, and other requirement) and hopefully beyond. It is essential that we develop effective and systematic burnout management programs for monitoring and preventing burnout in preparation against possible future outbreaks of infectious diseases.

The present researcher used a qualitative case study technique whereas a quantitative approach could confirm and support these results and build on the current study through further work on health workers’ burnout during COVID-19 in Jordanian hospitals. One limitation of this study is that the scope of research was confined to the outbreak of COVID-19 in one country. As such, it is necessary to increase the number of study contributors for comparison with cases in other countries. Besides, as most of the interviewees were male, future investigations need to include more female nurses, physicians, and pharmacist so as to examine gender characteristics. In addition, factors such as personality, coping strategies, and job attitude could be surveyed as additional influencing factors of burnout. Finally, the researchers also recommend that a mixed-method study design combining cross-sectional surveys and in-depth interviews be used for an in-depth inquiry into COVID-19-related burnout

## Data Availability

The datasets for this manuscript are not publicly available. The datasets obtained as a part of this will be available on request. Requests to access the datasets should be directed to faris.dahiyat@aau.ac.ae.
